# Respiratory effect of beta-blockers in people with asthma and cardiovascular disease: population-based nested case control study

**DOI:** 10.1186/s12916-017-0781-0

**Published:** 2017-01-27

**Authors:** Daniel R. Morales, Brian J. Lipworth, Peter T. Donnan, Cathy Jackson, Bruce Guthrie

**Affiliations:** 1 Quality, Safety & Informatics Group, Division of Population Health Sciences, School of Medicine, University of Dundee, Mackenzie Building, Dundee, DD2 4BF UK; 2 Scottish Centre for Respiratory Research, School of Medicine, University of Dundee, Dundee, UK; 3 Dundee Epidemiology and Biostatistics Unit, Division of Population Health Sciences, School of Medicine, University of Dundee, Dundee, UK; 4 School of Medicine, University of Central Lancashire, Preston, UK

**Keywords:** Asthma, Cardiovascular disease, Beta-blocker, Drug safety, Pharmacovigilance

## Abstract

**Background:**

Cardiovascular disease (CVD) is a common comorbidity in people with asthma. However, safety concerns have caused heterogeneity in clinical guideline recommendations over the use of cardioselective beta-blockers in people with asthma and CVD, partly because risk in the general population has been poorly quantified. The aim of this study was to measure the risk of asthma exacerbations with beta-blockers prescribed to a general population with asthma and CVD.

**Methods:**

Linked data from the UK Clinical Practice Research Datalink was used to perform nested case-control studies among people with asthma and CVD matched on age, sex and calendar time. Adjusted incidence rate ratios (IRR) were calculated for the association between oral beta-blocker use and moderate asthma exacerbations (rescue oral steroids) or severe asthma exacerbations (hospitalisation or death) using conditional logistic regression.

**Results:**

The cohort consisted of 35,502 people identified with active asthma and CVD, of which 14.1% and 1.2% were prescribed cardioselective and non-selective beta-blockers, respectively, during follow-up. Cardioselective beta-blocker use was not associated with a significantly increased risk of moderate or severe asthma exacerbations. Consistent results were obtained following sensitivity analyses and a self-controlled case series approach. In contrast, non-selective beta-blockers were associated with a significantly increased risk of moderate asthma exacerbations when initiated at low to moderate doses (IRR 5.16, 95% CI 1.83–14.54, *P* = 0.002), and both moderate and severe exacerbations when prescribed chronically at high dose (IRR 2.68, 95% CI 1.08–6.64, *P* = 0.033 and IRR 12.11, 95% CI 1.02–144.11, *P* = 0.048, respectively).

**Conclusions:**

Cardioselective beta-blockers prescribed to people with asthma and CVD were not associated with a significantly increased risk of moderate or severe asthma exacerbations and potentially could be used more widely when strongly indicated.

**Electronic supplementary material:**

The online version of this article (doi:10.1186/s12916-017-0781-0) contains supplementary material, which is available to authorized users.

## Background

Cardiovascular disease (CVD) is an important comorbidity in people with asthma who are up to three times more likely to develop CVD than people without [1]. Beta-blockers antagonise catecholamine-induced increases in heart rate, reduce blood pressure and improve left ventricular function, producing proven clinical benefits in people with CVD [2, 3]. Although beta-blockers may trigger exacerbations in susceptible people, they are still prescribed to some people with asthma possibly because benefit is perceived to outweigh risk [4, 5]. Evidence from clinical trials suggests that cardioselective beta-blockers are reasonably well tolerated in asthma with meta-analyses suggesting that adverse respiratory response to beta-blockers varies according to the degree of cardioselectivity, dose of administration and individual response [6, 7]. However, existing clinical trials have generally assessed acute beta-blocker exposure under controlled conditions in relatively selected individuals with asthma. It is therefore uncertain whether these results are generalisable to real world asthma populations.

Although certain asthma and cardiology guidelines now recommend that cardioselective beta-blockers may be used on a case-by-case basis in people with asthma, recommendations between clinical guidelines remain inconsistent with other national guidelines still recommending the avoidance of all beta-blockers in people with asthma [8–11]. This means that some people with asthma are being withheld beta-blockers despite strong clinical indications and proven benefits over their use [12]. These differences in recommendation may partly result from the risk of beta-blockers in people with asthma being poorly quantified, especially in real world populations were evidence is generally lacking. It is also increasingly recognised that some people may have the asthma/chronic obstructive pulmonary disease (COPD) overlap syndrome associated with a higher prevalence of CVD, making it particularly important to evaluate the safety of beta-blockers in asthma [13]. Evidence is therefore needed to evaluate the risk of beta-blockers in asthma from real life settings where routine beta-blocker prescribing occurs. The aim of this study was to measure whether oral beta-blocker exposure increases the risk of asthma exacerbations in a general population with active asthma and CVD.

## Methods

### Data source

Data were extracted from the Clinical Practice Research Datalink (CPRD), which contains electronic medical records from more than 680 UK general practices and more than 5 million people. CPRD contains linked data on patient demographics, prescriptions, diagnoses, hospitalisations and deaths. Diagnoses are recorded using Read Codes, a hierarchical thesaurus of coded clinical terms used in UK primary care [14]. CPRD is linked to hospital admissions via the Hospital Episode Statistics (HES) database, and to deaths via the Office for National Statistics (ONS) database. HES and ONS diagnoses are recorded using the International Classification of Disease (ICD10) coding system. General practices and patients within CPRD meet defined quality standards to contribute data, and diagnoses within CPRD have been shown to have high validity [15].

### Population

The cohort included people aged 18 years or above with actively treated asthma and actively treated CVD present in CPRD between January 1, 2000, and December 31, 2011. People with actively treated asthma and actively treated CVD were chosen so that controls were sampled from a more representative population. Subjects were eligible if they were permanently registered with a general practice for 1 year or more, were from general practices linked to HES and ONS databases, were defined by CPRD as being acceptable for use in research (meaning their data had met quality standards), or had a Read Code for asthma and a Read Code for a cardiovascular condition (ischaemic heart disease, chronic heart failure, cardiac arrhythmia, cerebrovascular disease, hypertension; code list contained within Additional file 1). People with Read Codes for COPD, bronchiectasis or restrictive lung disease were excluded in order to prevent misclassification bias as beta-blockers are likely to be better tolerated in this population.

Cohort entry was defined as the first prescription date for a CVD medicine issued on or after the latest of January 1, 2000, date of the first asthma medication, date of the patient’s 18th birthday, or before the date of the patient’s 80th birthday. Asthma medication consisted of inhaled short-acting beta2-agonists (SABA), inhaled corticosteroids (ICS), inhaled long-acting beta2-agonists (LABA), oral leukotriene antagonists, and oral methylxanthines [16]. Medication used for the management of CVD consisted of alpha blockers, beta-blockers (excluding those with propranolol because it is principally used for non-CVD conditions), calcium channel blockers, diuretics, nitrates and renin-angiotensin-system inhibitors [17]. The cohort was followed until either of the following occurred – an asthma event, deregistration from the general practice, 1 year following the last asthma medication (thereby censoring people with inactive asthma or asthma that had resolved), end of CVD medical treatment, or end of the study period (December 31, 2011). End of CVD medical treatment was defined by the last prescription date for a CVD medicine (plus a 90 day grace period) when 180 days had passed without any subsequent prescription for a CVD medicine.

### Study design and outcomes

The primary analysis was a nested case-control design used to more efficiently account for time-varying confounders and drug exposure [18]. The nested case-control design assesses the risk of exposure versus non-exposure among cases and controls and it is normal for cases to appear ‘sicker’ than controls [19]. Two nested case-control studies were performed evaluating (1) moderate asthma exacerbations and (2) severe asthma exacerbations. Severe asthma exacerbations were defined as a hospitalisation for asthma (defined as admissions with ICD codes for asthma recorded as the primary reason for hospitalisation) or death from asthma. Moderate asthma exacerbations were identified by receipt of rescue oral steroids in primary care, defined as oral prednisolone prescriptions of less than 2 weeks duration using ≥ 5 mg strength tablets. People with non-rescue oral steroids were excluded from this analysis to prevent outcome misclassification bias. For each outcome, the date of the first asthma event was the index date for case subjects.

### Control selection

Up to 10 controls were randomly selected and matched to each case on age decile, gender and calendar year of cohort entry using incidence density sampling. The risk set date for controls was the index date for cases. With incidence density sampling, ‘controls’ are a selection of person-moments from individuals who have not experienced the event at the index date [19]. In this regard, controls may be selected more than once, and people who subsequently become cases may be selected as controls at earlier time points. Two cases of severe asthma exacerbation (0.3%) and eight cases of moderate asthma exacerbation (0.2%) were initially unmatched, but were included matched on sex and calendar year of cohort entry only and sensitivity analysis was performed excluding these cases.

### Exposures

Exposure to beta-blockers used for the management of CVD was measured by prescriptions issued prior to the index date. Beta-blocker exposure was categorised into current acute exposure (defined as a prescription issued within 60 days of the index date and no previous prescription issued in days 61–365 before the index date); current chronic exposure (defined as a prescription issued within 60 days of the index date and one or more prescriptions issued in days 61–365 before the index date); and no exposure when there was no prescription issued in a 60-day risk window before the index dates. Among current users, beta-blocker exposure was evaluated by cardioselectivity and dose. Dose was stratified into low to moderate daily dose and high daily dose. High dose beta-blocker exposure was defined by daily doses greater than the following: acebutolol 200 mg, atenolol 50 mg, bisoprolol 5 mg, carvedilol 25 mg, celiprolol 200 mg, metoprolol 100 mg, nadolol 80 mg, oxprenolol 80 mg, pindolol 10 mg, sotalol 160 mg, and timolol 10 mg.

### Confounders

In recognition of the stepwise approach to asthma management and to account for the severity of asthma, analyses were adjusted for current use of asthma medication defined as a prescription for either of SABA, ICS, LABA, leukotriene antagonists or methylxanthines issued within 90 days of the index date. As a sensitivity analysis, ICS was modelled by fluticasone-equivalent doses, categorised as high (fluticasone ≥ 1000 μg/day), moderate (500–999 μg/day) and low (<500 μg/day) dose according to their relative topical potency [10]. Additional risk adjustment was performed for respiratory tract infection diagnosed within 90 days of the index date, prior hospitalisation for asthma, prescription for CVD medicine use in the year prior to the index date (consisting of prescriptions for alpha blockers, calcium channel blockers, diuretics, nitrates and renin-angiotensin system medicine), exact age, smoking status, body mass index, index of multiple deprivation, Charlson comorbidity index, and attendance at a primary care asthma review in the year prior to the index date.

### Data analysis

Conditional logistic regression was used to calculate odds ratios for the association between asthma exacerbations and beta-blocker exposure. Using an incidence density sampling approach, odds ratios represented unbiased estimators of incidence rate ratios (IRR). Adjusted rate differences were calculated for significant associations providing an absolute measure of effect [19]. Multiple imputation was used to impute missing data on height, weight and smoking status. The imputation model included all variables relating to clinical characteristics, asthma events, medication and beta-blocker exposure. Multiple imputation used fully conditional specification, with linear regression for continuous variables and logistic regression for categorical variables with five imputations analysed using Rubin’s rules [20]. Analysis was carried out using SPSSv21 and STATAv13.

### Sensitivity analyses and secondary self-controlled case series analysis

Sensitivity analyses were performed for the primary analysis, namely modelling ICS by dose, excluding cases not originally matched on age, people hospitalised within the risk period (assessing for potential immeasurable time bias) [21], people over the age of 40 years who smoked (assessing for potential misclassification with undiagnosed or unrecorded COPD), and using a complete case analysis and a 30- and 90-day risk window (assessing whether risk attenuated over time and because the exact date patients started taking their medication was unknown). Furthermore, we evaluated the association between nitrate exposure and risk of asthma exacerbations in the form of a negative control.

As a secondary analysis a self-controlled case series with adjustment for time-varying confounders was performed to further measure the safety of acute cardioselective beta-blocker exposure and the risk of moderate asthma exacerbations [16]. In contrast to the nested case control study, the self-controlled case series is a within-person design whereby the patient acts as their own control, controlling for all fixed-confounders. Incidence rate ratios were calculated using conditional Poisson regression. Full details of the self-controlled case series approach are contained in Additional file 2.

## Results

The cohort consisted of 35,502 people with actively treated asthma and CVD (mean age 60.1 years, 59.7% women). During follow-up, cardioselective beta-blockers were prescribed to 5017 patients (14.1%) and non-selective beta-blockers were prescribed to 407 patients (1.2%). Cardioselective beta-blocker exposure consisted mainly of atenolol (7.9%) and bisoprolol (5.4%), whilst non-selective beta-blocker exposure consisted mainly of sotalol (0.6%) and carvedilol (0.4%). A total of 608 severe asthma exacerbations (incidence 4.4 per 1000 person-years) and 4234 moderate asthma exacerbations (incidence 50.4 per 1000 person years) occurred during follow-up (mean 3.5 years). The 608 cases of severe asthma exacerbation were matched to 6048 controls and the 4234 cases of moderate asthma exacerbation were matched to 41,881 controls, selected from the cohort risk-sets (Table 1). Cases and controls were well matched on age, sex and duration of follow-up.

**Table 1:** Characteristics of cases and controls for severe and moderate asthma exacerbations

	Severe asthma exacerbations		Moderate asthma exacerbation	
	Cases	Controls	Cases	Controls
Number of people	608	6048	4234	41,881
Female sex, no. (%)	428 (70.4)	4261 (70.5)	2815 (66.5)	27,898 (66.6)
Age (years), mean ± SD	62.4 ± 13.4	62.5 ± 13.3	62.8 ± 11.8	62.9 ± 11.7
Years of follow-up, mean ± SD	2.9 ± 2.9	2.9 ± 2.9	2.0 ± 2.5	2.0 ± 2.5
Asthma medication use,^a^ no. (%)				
SABA	482 (79.3)	3631 (60.0)	2809 (66.3)	22,916 (54.7)
ICS	492 (80.1)	4336 (71.7)	3060 (72.3)	27,236 (65.0)
LABA	339 (55.8)	1905 (31.5)	1407 (33.2)	9975 (23.8)
Leukotriene antagonists	67 (11.0)	193 (3.2)	108 (2.6)	731 (1.8)
Methylxanthines	52 (8.6)	107 (1.7)	80 (1.9)	564 (1.4)
Oral corticosteroids	270 (44.4)	417 (6.9)	n/a	n/a
Cardiac medication use,^b^ no. (%)				
Alpha blockers	68 (11.2)	590 (9.8)	395 (9.3)	3774 (9.0)
Beta-blockers	47 (7.7)	533 (8.8)	425 (10.0)	4628 (11.1)
Calcium channel blockers	293 (48.2)	2755 (45.6)	1775 (41.9)	18,708 (44.7)
Diuretics	333 (54.8)	3210 (53.1)	2185 (51.6)	21,240 (50.7)
Nitrates	97 (16.0)	653 (10.8)	495 (11.7)	4760 (11.4)
Renin angiotensin system inhibitors	404 (66.5)	3973 (65.7)	2679 (63.3)	26344 (62.3)
Charlson comorbidity index, ± SD	2.0 ± 1.5	1.9 ± 1.4	1.8 ± 1.3	2.0 ± 1.5
Body mass index, ± SD	31.0 ± 7.0	30.1 ± 6.3	30.2 ± 6.3	29.7 ± 6.0
Smoking status, no. (%)				
Current smoker	63 (10.4)	566 (9.4)	444 (10.5)	4132 (9.9)
Non-smoker	516 (84.9)	5314 (87.9)	3664 (86.5)	36,256 (86.5)
Missing	29 (4.8)	168 (2.8)	126 (3.0)	1493 (3.6)
Respiratory tract infection,^a^ no. (%)	110 (18.1)	398 (6.6)	597 (14.1)	2001 (4.8)
Ever hospitalised for asthma, no. (%)	84 (13.8)	120 (2.0)	115 (2.7)	602 (1.5)
Primary care asthma review,^b^ no. (%)	283 (46.6)	2917 (48.2)	2104 (49.7)	18,905 (45.1)

^a^In the 90 days prior to the index date

^b^In the year prior to the index date

*No.* number, *SD* standard deviation, *SABA* short-acting beta2-agonists, *ICS* inhaled corticosteroids, *LABA* long-acting beta2-agonists, *n/a* not applicable

### Cardioselective beta-blocker exposure

Incidence rate ratios for moderate and severe asthma exacerbations associated with cardioselective beta-blocker exposure according to dose are presented in Table 2. Cardioselective beta-blocker exposure was not significantly associated with an increased risk of moderate asthma exacerbations (IRR 0.97, 95% CI 0.85–1.11, *P* = 0.658) or of severe asthma exacerbations (IRR 0.87, 95% CI 0.57–1.35, *P* = 0.540). Risk of moderate asthma exacerbations was not significantly increased with low- to moderate-dose cardioselective beta-blocker exposure (IRR 0.96, 95% CI 0.83–1.10, *P* = 0.544) or high-dose cardioselective beta-blocker exposure (IRR 1.08, 95% CI 0.82–1.42, *P* = 0.600). Similarly, risk of severe asthma exacerbations was not significantly increased with low- to moderate-dose cardioselective beta-blocker exposure (IRR 0.85, 95% CI 0.53–1.36, *P* = 0.501) or high-dose cardioselective beta-blocker exposure (IRR 0.96, 95% CI 0.33–2.84, *P* = 0.943). When further evaluated by dose and duration of exposure, risk of moderate or severe asthma exacerbations was not significantly increased with either acute or chronic cardioselective beta-blocker exposure (Table 3).

**Table 2:** Incidence rate ratios for the association between beta-blocker exposure and asthma exacerbations by dose

	Cardioselective beta-blockers						Non-selective beta-blockers					
	Exposed	Exposed	Crude	Adjusted			Exposed	Exposed	Crude	Adjusted		
	cases^a^	controls^a^	IRR	IRR	95% CI	*P* value	cases^a^	controls^a^	IRR	IRR	95% CI	*P* value
Any exposure												
Severe exacerbation	27	466	0.72	0.87	0.57–1.35	0.540	9	51	1.29	1.66	0.53–5.35	0.398
Moderate exacerbation	357	3956	0.89	0.97	0.85–1.11	0.658	35	309	1.33	1.41	0.95–2.08	0.088
Low dose												
Severe exacerbation	23	388	0.70	0.85	0.53–1.36	0.501	8	44	1.04	1.19	0.31–4.53	0.799
Moderate exacerbation	283	3256	0.87	0.96	0.83–1.10	0.544	29	271	1.19	1.24	0.80–1.91	0.336
High dose												
Severe exacerbation	4	82	0.85	0.96	0.33–2.84	0.943	1	7	5.00	12.11	1.02–144.11	0.048
Moderate exacerbation	79	733	0.99	1.08	0.82–1.42	0.600	6	39	2.50	2.67	1.08–6.62	0.034

^a^Exposed cases/controls, exposed within the 60 day risk window

*IRR* Incidence Rate Ratios

Adjusted for asthma medication use in the 90 days prior to the index date; respiratory tract infection in the 90 days prior to the index date; prior hospitalization for asthma; type of CVD medicine use in the year prior to the index date; exact age; smoking status; body mass index; social deprivation; Charlson comorbidity index; and primary care asthma review in the year prior to the index date

**Table 3:** Incidence rate ratios for the association between beta-blocker exposure and asthma exacerbations by dose and duration of exposure

	Cardioselective beta-blockers						Non-selective beta-blockers					
	Exposed	Exposed	Crude	Adjusted			Exposed	Exposed	Crude	Adjusted		
	cases^a^	controls^a^	IRR	IRR	95% CI	*P* value	cases^a^	controls^a^	IRR	IRR	95% CI	*P* value
Low to moderate dose												
Acute												
Severe exacerbation^b^	4	28	1.41	1.47	0.44–4.97	0.532	0	2	–	–	–	–
Moderate exacerbation	19	255	1.02	1.04	0.64–1.70	0.865	6	12	5.19	5.16	1.83–14.54	0.002
Chronic												
Severe exacerbation^b^	19	360	0.63	0.81	0.48–1.35	0.409	8	42	1.11	1.22	0.32–4.67	0.773
Moderate exacerbation	264	3031	0.86	0.95	0.82–1.10	0.517	23	259	0.86	0.99	0.60–1.62	0.954
High dose												
Acute												
Severe exacerbation^b^	1	6	1.67	2.76	0.32–23.78	0.347	0	0	–	–	–	–
Moderate exacerbation	2	51	0.49	0.55	0.13–2.31	0.416	0	0	–	–	–	–
Chronic												
Severe exacerbation^b^	7	63	0.73	0.82	0.24–2.82	0.754	1	7	5.00	12.04	1.01–143.48	0.049
Moderate exacerbation	77	682	1.03	1.11	0.84–1.47	0.339	6	39	2.50	2.68	1.08–6.64	0.033

^a^Exposed cases/controls, exposed within the 60 day risk window

^b^Severe asthma exacerbations associated with acute non-selective beta-blocker exposure inestimable due to lack of exposure *IRR* Incidence Rate Ratios

Adjusted for asthma medication use in the 90 days prior to the index date; respiratory tract infection in the 90 days prior to the index date; prior hospitalization for asthma; type of CVD medicine use in the year prior to the index date; exact age; smoking status; body mass index; social deprivation; Charlson comorbidity index; and primary care asthma review in the year prior to the index date. Empty cells (–), inestimable due to lack of corresponding beta-blocker exposure among cases and controls

### Non-selective beta-blocker exposure

High-dose non-selective beta-blocker exposure was associated with a significantly increased rate of moderate asthma exacerbations (IRR 2.67, 95% CI 1.08–6.62, *P* = 0.034) and of severe asthma exacerbations (IRR 12.11, 95% CI 1.02–144.11, *P* = 0.048), with adjusted rate differences of 63.2 (95% CI 25.7–156.4) and 27.0 (95% CI 2.3–337.7) per 1000 person-years, respectively (Table 2). For severe asthma exacerbations, high-dose non-selective beta-blocker exposure consisted entirely of chronic exposure. In contrast, low- to moderate-dose non-selective beta-blocker exposure was not associated with a significantly increased relative incidence of moderate (IRR 1.24, 95% CI 0.80–1.91, *P* = 0.336) or severe asthma exacerbations (IRR 1.19, 95% CI 0.31–4.53, *P* = 0.799).

When evaluated by dose and duration of exposure, the relative incidence of moderate asthma exacerbations was significantly increased with acute low- to moderate-dose non-selective beta-blocker exposure (IRR 5.16, 95% CI 1.83–14.54, *P* = 0.002), with an adjusted rate difference of 134.5 (95% CI 25.9–370.6) per 1000 person years (Table 3). In contrast, chronic low- to moderate-dose non-selective beta-blocker exposure was not associated with an increased risk of moderate (IRR 0.99, 95% CI 0.60–1.62, *P* = 0.954) or severe (IRR 1.22, 95% CI 0.32–4.67, *P* = 0.773) asthma exacerbations.

#### Sensitivity analyses and secondary self-controlled case series analysis

Sensitivity analyses for the primary analysis were consistent with the main findings, showing no significantly increased risk of moderate or severe asthma exacerbations associated with cardioselective beta-blocker exposure, an increased risk of moderate asthma exacerbations associated with high-dose and acute low- to moderate-dose non-selective beta-blocker exposure, and an increased risk of severe asthma exacerbations associated with high-dose non-selective beta-blocker exposure (Additional files 3 and 4). When nitrate exposure was used as a negative control in the primary analysis, there was no significant increased risk of moderate or severe asthma exacerbations associated with acute or chronic nitrate exposure (Table 4).

**Table 4:** Risk of moderate and severe asthma exacerbations using a negative control with nitrate exposure

	Nitrates					
	Exposed	Exposed	Crude	Adjusted		
	cases^a^	controls^a^	IRR	IRR	95% CI	*P* value
Any exposure						
Severe exacerbation	65	408	1.68	1.19	0.86–1.65	0.287
Moderate exacerbation	329	2896	1.14	1.10	0.97–1.25	0.131
Acute exposure						
Severe exacerbation	5	45	1.16	1.36	0.50–3.69	0.550
Moderate exacerbation	40	343	1.16	1.14	0.81–1.59	0.600
Chronic exposure						
Severe exacerbation	60	363	1.75	1.18	0.84–1.65	0.463
Moderate exacerbation	289	2553	1.14	1.10	0.96–1.26	0.172

^a^Exposed cases/controls, exposed within the 60 day risk window

*IRR* Incidence Rate Ratios

Adjusted for asthma medication use in the 90 days prior to the index date; respiratory tract infection in the 90 days prior to the index date; hospitalization for asthma in the year prior to the index date; type of CVD medicine use in the year prior to the index date; exact age; smoking status; body mass index; social deprivation; Charlson comorbidity index; and primary care asthma review in the year prior to the index date

The self-controlled case series assessing the risk associated with acute cardioselective beta-blocker exposure produced consistent findings with no significantly increased risk of moderate asthma exacerbations when using a 30-, 60- or 90-day acute risk window following cardioselective beta-blocker initiation (IRR 1.01, 95% CI 0.66–1.54 for a 30-day risk window, IRR 0.99, 95% CI 0.72–1.38 for a 60-day risk window, and IRR 0.93, 95% CI 0.69–1.25 for a 90-day risk window) (please see Additional file 2 for further details).

## Discussion

Although managing comorbidity is the norm in modern medicine, clinical uncertainty still exists around whether to prescribe cardioselective beta-blockers to people with asthma and CVD. Our findings suggest that the adverse respiratory response to beta-blockers in asthma depends partly upon cardioselectivity, dose and duration of exposure. Among our population with active asthma and CVD, oral cardioselective beta-blocker exposure was not associated with a significantly increased risk of asthma exacerbations. In contrast, oral non-selective beta-blocker exposure was associated with a significantly increased risk of asthma exacerbations when initiated at low to moderate doses, and when prescribed chronically at high doses.

Apparent differences in risk between acute and chronic low- to moderate-dose oral non-selective beta-blocker exposure could be due to attenuation of risk associated with beta2-adrenoceptor up-regulation, as suggested by studies evaluating chronic dosing effects of oral beta-blockers in asthma, or survival bias whereby people are more likely to receive longer-term therapy if they tolerate acute exposure [22]. Studies investigating chronic oral non-selective beta-blocker exposure in asthma have typically used selected populations of well controlled asthmatics initiating oral non-selective beta-blockers at low dose, using inhaled muscarinic antagonist cover to prevent bronchoconstriction from unopposed cholinergic tone [23]. Our previous meta-analysis of randomised controlled trials demonstrated that acute oral non-selective beta-blocker exposure caused mean falls in forced expiratory volume in one second (FEV1) of 10%, an increase in respiratory symptoms affecting one in 13 people, and falls in FEV1 of ≥ 20% affecting one in nine people with asthma [6]. It has also been demonstrated that acute non-selective beta-blocker eye drop exposure caused mean falls in FEV1 of 11% and falls in FEV1 of ≥ 20% affecting one in three people with asthma in clinical trials, and an increased risk of moderate asthma exacerbations in routine practice [24]. Although it appears that some people with asthma do tolerate non-selective beta-blockers, risk of bronchoconstriction is therefore much greater, and response to SABA rescue therapy is significantly blunted with fatal cases having been reported following treatment of myocardial infarction [6, 25]. Given recommendations for rapid treatment of myocardial infarction, in such instances, it is important not to overlook proper assessment of patients to identify those with comorbid asthma [26]. Findings from our current study therefore support established recommendations that non-selective beta-blockers should not be prescribed for the management of CVD in people with asthma.

Our previous meta-analysis of randomised controlled trials also demonstrated that acute oral cardioselective beta-blockade (consisting largely of moderate- to high-dose exposure) caused asymptomatic mean falls in FEV1 of 7%, and asymptomatic falls in FEV1 of ≥ 20% affecting one in eight people with asthma. Our real world observational study found that oral cardioselective beta-blocker use in people with active asthma and CVD was not associated with a significantly increased risk of moderate or severe asthma exacerbations [6]. Therefore, these findings do not support recommendations present in some asthma and CVD guidelines that all beta-blockers should be avoided in people with asthma when strong clinical indications exist [10, 11]. In this regard, the overall benefit-risk of using beta-blockers in people with asthma should be taken into account. This study also suggests that cardioselective beta-blockers should not be routinely discontinued in people with asthma if already established on cardioselective beta-blockers providing they are appropriately indicated. This is important for prescribing safety interventions that may class and target the use of cardioselective beta-blockers in people with asthma as high risk [27].

Although no significant increase in exacerbations occurred with acute high dose cardioselective beta-blocker exposure, confidence intervals were wide and our previous meta-analysis of randomised controlled trials highlighted a dose–response relationship may occur with acute cardioselective beta-blocker exposure [6]. As such, we cannot exclude the possibility that a higher risk of exacerbation exists in such comparisons. For this reason, if cardioselective beta-blockers are to be considered in people with asthma, they should realistically only be initiated at low dose with gradual dose titration, ensuring the availability of reliever therapy that is still reasonably effective during acute cardioselective beta-blockade should symptoms develop [6]. Regardless of comorbid asthma, initiating beta-blockers at low dose with gradual dose titration should be routinely recommended to prevent episodes of hypotension and bradycardia. Prior to considering cardioselective beta-blockers in people with asthma and CVD, however, a benefit–risk assessment would be prudent ideally taking into account patient preference.

The mean age of patients in our study was 62 years such that some patients might have asthma-COPD overlap syndrome, raising the important related question as to the safety of beta-blockers in COPD where the burden of CVD is higher. Current evidence suggests that patients with COPD taking long term beta-blockers have reduced exacerbations and reduced mortality [28]. Despite this observation, beta-blockers appear to be underused in people with COPD and heart failure [29, 30]. Patients with COPD may be less likely to bronchoconstrict from beta-blockade because of greater fixed airflow obstruction and attenuated beta2-adrenoceptor responsiveness, which may vary depending on the asthmatic component. Nonetheless, our data are reassuring for patients taking cardioselective beta-blockers for the management of CVD irrespective of having pure asthma or asthma-COPD overlap syndrome.

This study has several limitations. First, not all types of beta-blocker exposure could be properly assessed, including acute high dose non-selective beta-blocker exposure were risk is likely to be greater. Given current guideline recommendations surrounding the use of non-selective beta-blockers in asthma, it is unsurprising that acute high dose non-selective beta-blocker exposure in people with asthma and CVD was rare. However, our principle aim was to evaluate cardioselective beta-blocker exposure to better inform their use in people with asthma and CVD, where there is an unmet need. In this context, identifying an increased risk from non-selective beta-blocker exposure helps to act as a positive control. Second, we cannot exclude the possibility that some people had a degree of fixed air-flow obstruction because lung function data was not routinely available and residual confounding may exist, which is possible in all observational studies. However, several sensitivity analyses were conducted with results in keeping with the main analysis. Third, prescription dates were used as a proxy for exposure and it is uncertain exactly when patients commenced their medication. Fourth, we cannot exclude a degree of selection bias among asthmatics prescribed beta-blockers in our cohort potentially affecting the generalisability of results to all people with asthma. However, the crude prevalence of beta-blocker prescribing between cases and controls was very similar, and some people with asthma were still prescribed non-selective beta-blockers shown to be associated with a significantly increased risk of asthma exacerbations supporting our findings. Fifth, observational studies may be prone to bias. Our primary analysis used a nested case control study, a between-person study design. We therefore performed a self-controlled case series, a within person design were the patient acts as their own control, assessing acute cardioselective beta-blocker exposure with consistent results. Furthermore, we evaluated a negative control using nitrate exposure. Finally, outcomes and exposures relied upon electronic prescribing and coding, and it remains possible that not all of these were identified. Nevertheless, hospital discharges are routinely captured electronically in the UK and almost all community prescriptions are issued electronically from general practice, including those initiated by hospital specialists.

## Conclusions

In conclusion, this study suggests that the adverse respiratory response from beta-blockers in people with asthma and CVD varies according to cardioselectivity, dose and duration of exposure. In contrast to oral non-selective beta-blockers, oral cardioselective beta-blocker exposure was not associated with a significantly increased risk of asthma exacerbations and should potentially be considered more widely in people with strong clinical indications.

## Additional files

Additional file 1: Table S1. Read Codes identifying people with asthma and cardiovascular disease in the actively treated asthma and CVD cohort. (DOCX 12 kb)
Additional file 2: Table S2. Incidence rate ratios for cardioselective beta-blocker exposure and moderate asthma exacerbations in the self-controlled case series. (DOCX 16 kb)
Additional file 3: Table S3. Sensitivity analyses for cardioselective beta-blocker exposure and asthma exacerbations. (DOCX 20 kb)
Additional file 4: Table S4. Sensitivity analyses for non-selective beta-blocker exposure and asthma exacerbations. (DOCX 17 kb)

## Abbreviations

CI: confidence interval
COPD: chronic obstructive pulmonary disease
CVD: cardiovascular disease
FEV1: forced expiratory volume in 1 second
HES: hospital episodes statistics
ICD: International Classification of Disease
ICS: inhaled corticosteroid
IRR: incidence rate ratio
LABA: long-acting beta2-agonist
ONS: Office for National Statistics
SABA: short-acting beta2-agonist
