# Supplementary material for: Respiratory effect of beta-blockers in people with asthma and cardiovascular disease: population-based nested case control study
Source: BMC Med. 2017 Jan 27;15:18. doi: 10.1186/s12916-017-0781-0 (PMC5270217; doi:10.1186/s12916-017-0781-0)
Supplement: Additional file 1: Table S1. — Read Codes identifying people with asthma and cardiovascular disease in the actively treated asthma and CVD cohort. (DOCX 12 kb) [file 12916_2017_781_MOESM1_ESM.docx]

**ADDITIONAL FILE 1: BETA-BLOCKERS IN PEOPLE WITH ASTHMA AND CVD**

**Table S1. Read Codes identifying people with asthma and cardiovascular disease in the actively treated asthma and CVD cohort.**

| **Read Codes** | |
| --- | --- |
| **Asthma** | **Cardiovascular disease** |
| H33..00, 663..11,  14B4.00, H333.00,  663V.00, H33zz00, H330.00, H33..11, 173A.00, 663n.00, H331.00, H33z100, H33z200, 663v.00, H330.11, H33z.00, 1O2..00, H330011, 663V100, H330.14, H331.11, 663N.00, 663P.00, H33z.11, 663V000, 9Q21.00, 663J.00, 663p.00, H330.13, H33z011, 663j.00, H33z000, H33zz11, 663V200, H330000, H330111, 663d.00, H330z00, H331z00, H33zz12, H33zz13 | G3...00, G3...11, G3...12, G3...13, G30..11, G30..12, G30..14, G301z00, G305.00, G306.00, G311.00, G311.11, G311.13, G311.14, G311100, G311200, G311300, G311400, G311500, G311z00, G312.00, G31y.00, G31y000, G31y200, G31y300, G31yz00, G32..11, G33..00, G330.00, G330000, G330z00, G33z.00, G33z000, G33z300, G33z400, G33z500, G33z600, G33z700, G33zz00, G340.00, G340.11, G340.12, G340000, G340100, G342.00, G343.00, G344.00, G34y.00, G34y000, G34y100, G34yz00, G34z.00, G34z000, G3z..00, G30..00, G30..15, G30..16, G30..17, G300.00, G301.00, G301000, G301100, G302.00, G303.00, G304.00, G307.00, G307000, G307100, G308.00, G309.00, G30B.00, G30X.00, G30X000, G30y.00, G30y000, G30y200, G30yz00, G30z.00, G31y100, G32..00, G32..12, G35..00, G350.00, G351.00, G35X.00, G38..00, G381.00, G384.00, G2...00, G2...11, G20..00, G20..11, G200.00, G201.00,G202.00, G203.00, G20z.00, G20z.11, G24..00, G240.00, G241.00, G244.00, G24z.00, G24z000, G24zz00, G2z..00, Gyu2.00, G61..00, G61..11, G61..12, G63y000, G63y100, G64..00, G64..11, G64..12, G64..13, G65..00, G65..12, G65..13, G656.00, G65y.00, G65z.00, G65z100, G65zz00, G676000, G6W..00, G6X..00, Gyu6300,Gyu6400, Gyu6500, Gyu6600, Gyu6G00, G73..00, G73..11, G73..12, G73..13, G733.00, G73y.00, G73yz00, G73z.00, G73z000, G73z011, G73z012, G73zz00, Gyu7400, 327..00, 328..00, G57..11, G573.00, G573000, G573100, G573200, G573300, G573400, G573500, G573z00, G57yA00, G65..11, Gyu5a00, 585f.00, 585g.00, 662f.00, 662g.00, 662h.00, 662i.00, G1yz100, G58..00, G58..11, G5yy900, G5yyA00 |
