# Supplementary material for: Respiratory effect of beta-blockers in people with asthma and cardiovascular disease: population-based nested case control study
Source: BMC Med. 2017 Jan 27;15:18. doi: 10.1186/s12916-017-0781-0 (PMC5270217; doi:10.1186/s12916-017-0781-0)
Supplement: Additional file 2: Table S2. — Incidence rate ratios for cardioselective beta-blocker exposure and moderate asthma exacerbations in the self-controlled case series. (DOCX 16 kb) [file 12916_2017_781_MOESM2_ESM.docx]

**ADDITIONAL FILE 2: BETA-BLOCKERS IN PEOPLE WITH ASTHMA AND CVD**

**SELF-CONTROLLED CASE SERIES SUPPLEMENTARY METHODS**

**Self-controlled case series study and risk periods**

A secondary analysis evaluating the risk of moderate asthma exacerbations with acute cardioselective beta-blocker exposure was performed using a self-controlled case series. Only moderate asthma exacerbations were included because no severe asthma exacerbations occurred during the self-controlled case series observation period. A 360 day observation period was used commencing 180 days prior to incident cardioselective beta-blocker exposure (defined by the date of the first cardioselective beta-blocker prescription in people with at least 1 year’s follow-up prior to receipt). A 30 day pre-risk period was excluded from the analysis to account for potential event-dependent exposures (17). Oral steroid prescriptions issued within eight days of each other were considered treatment for the same asthma exacerbation and counted only once. A 30 day, 60 day and 90 day acute risk period commencing from the date of the incident cardioselective beta-blocker prescription was used to compare with results from the nested case control study. Exposure beyond each acute risk period was defined as chronic exposure. End of cardioselective beta-blocker exposure was calculated using the quantity and frequency dosing instructions for each cardioselective beta-blocker prescription.

**Self-controlled case series confounder adjustment and data analysis**

The SCCS is a within-person design with the patient acting as their own control, thereby controlling for all time-fixed confounding. To control for potential time-varying confounding, the observation period was restricted to 360 days and adjustment made for the following time-varying exposures: ICS; LABAs; leukotriene antagonists; methlyxanthines; number of SABA prescriptions; and seasonal variation. Age was not a significant time-varying confounder in this analysis because of the short study period. The duration of any hospitalizations occurring during the study period were calculated and this person time subtracted from each corresponding exposure group to prevent immeasurable time bias. The self-controlled case series was analysed using conditional Poisson regression producing incidence rate ratios (IRRs) and 95% confidence intervals (17).

**SELF-CONTROLLED CASE SERIES SUPPLEMENTARY RESULTS**

Of 948 eligible patients with incident cardioselective beta-blocker exposure, only 168 patients (17.7%) experienced ≥1 moderate asthma exacerbation during the self-controlled case series observation period (mean age 66.2 years, 66.7% women). The number of events, total person time and relative incidence of moderate asthma exacerbations for the baseline period, acute risk periods and the chronic risk periods are shown in supplementary table S4. There was no significant increase in the relative incidence of moderate asthma exacerbations with acute cardioselective beta-blocker exposure irrespective of risk period duration evaluated.

**Table S2. Incidence rate ratios for cardioselective beta-blocker exposure and moderate asthma exacerbations in the self-controlled case series.**

| **Risk Period** | **Person time**  **(Days)** | **Events** | **Crude**  **IRR (95%CI)** | **Adjusted**  **IRR (95%CI)** | **p-value** |
| --- | --- | --- | --- | --- | --- |
| 30 day risk period |  |  |  |  |  |
| - Baseline | 29583 | 163 | Reference | Reference | - |
| - Acute | 4915 | 27 | 0.97 (0.64-1.45) | 1.01 (0.66-1.54) | 0.963 |
| - Chronic | 19977 | 109 | 0.94 (0.73-1.21) | 0.94 (0.71-1.23) | 0.637 |
| 60 day risk period |  |  |  |  |  |
| - Baseline | 29075 | 161 | Reference | Reference | - |
| - Acute | 9955 | 53 | 0.95 (0.70-1.30) | 0.99 (0.72-1.38) | 0.970 |
| - Chronic | 15445 | 85 | 0.94 (0.71-1.24) | 0.93 (0.69-1.25) | 0.622 |
| 90 day risk period |  |  |  |  |  |
| - Baseline | 28203 | 158 | Reference | Reference | - |
| - Acute | 14930 | 75 | 0.89 (0.68-1.18) | 0.93 (0.69-1.25) | 0.628 |
| - Chronic | 11321 | 66 | 1.00 (0.74-1.35) | 0.98 (0.70-1.37) | 0.915 |

IRR = incidence rate ratios. N=168 exposed cases with asthma and cardiovascular disease. Adjusted for seasonal variation and use of: inhaled corticosteroids; long-acting beta2-agonists; methylxanthines; leukotriene antagonists; and short-acting beta2-agonists.
