# Supplementary material for: Respiratory effect of beta-blockers in people with asthma and cardiovascular disease: population-based nested case control study
Source: BMC Med. 2017 Jan 27;15:18. doi: 10.1186/s12916-017-0781-0 (PMC5270217; doi:10.1186/s12916-017-0781-0)
Supplement: Additional file 3: Table S3. — Sensitivity analyses for cardioselective beta-blocker exposure and asthma exacerbations. (DOCX 20 kb) [file 12916_2017_781_MOESM3_ESM.docx]

**ADDITIONAL FILE 3: BETA-BLOCKERS IN PEOPLE WITH ASTHMA AND CVD**

**Table S3. Sensitivity analyses for cardioselective beta-blocker exposure and asthma exacerbations.**

|  | **Any**  **exposure** | | **High dose**  **exposure** | | **Low-moderate**  **dose exposure** | | **Acute**  **exposure** | | **Chronic**  **exposure** | |
| --- | --- | --- | --- | --- | --- | --- | --- | --- | --- | --- |
|  | **IRR** | **95% CI** | **IRR** | **95% CI** | **IRR** | **95% CI** | **IRR** | **95% CI** | **IRR** | **95% CI** |
| **Inhaled corticosteroids by dose** |  |  |  |  |  |  |  |  |  |  |
| - Severe asthma exacerbation | 0.91 | 0.59-1.40 | 1.03 | 0.35-3.03 | 0.88 | 0.55-1.40 | 2.15 | 0.70-6.56 | 0.81 | 0.50-1.29 |
| - Moderate asthma exacerbation | 0.97 | 0.85-1.11 | 1.08 | 0.82-1.42 | 0.96 | 0.83-1.10 | 0.91 | 0.53-1.57 | 0.97 | 0.85-1.11 |
| **Hospitalised in risk window** |  |  |  |  |  |  |  |  |  |  |
| - Severe asthma exacerbation | 1.00 | 0.61-1.62 | 1.25 | 0.42-3.77 | 0.95 | 0.56-1.61 | 2.78 | 0.77-10.12 | 0.88 | 0.52-1.49 |
| - Moderate asthma exacerbation | 0.97 | 0.85-1.11 | 1.09 | 0.82-1.45 | 0.96 | 0.83-1.11 | 0.83 | 0.43-1.60 | 0.98 | 0.85-1.12 |
| **Smokers over 40 years** |  |  |  |  |  |  |  |  |  |  |
| - Severe asthma exacerbation | 0.82 | 0.50-1.34 | 0.80 | 0.23-2.74 | 0.82 | 0.48-1.39 | 2.96 | 0.88-9.97 | 0.68 | 0.39-1.19 |
| - Moderate asthma exacerbation | 1.03 | 0.90-1.18 | 1.08 | 0.80-1.45 | 1.03 | 0.89-1.19 | 1.00 | 0.58-1.74 | 1.03 | 0.89-1.18 |
| **Unmatched on age** |  |  |  |  |  |  |  |  |  |  |
| - Severe asthma exacerbation | 0.92 | 0.59-1.43 | 1.05 | 0.36-3.09 | 0.89 | 0.56-1.43 | 2.24 | 0.70-7.11 | 0.82 | 0.51-1.34 |
| - Moderate asthma exacerbation | 0.99 | 0.87-1.13 | 1.11 | 0.85-1.47 | 0.97 | 0.85-1.12 | 0.92 | 0.54-1.58 | 0.99 | 0.87-1.13 |
| **Complete case analysis** |  |  |  |  |  |  |  |  |  |  |
| - Severe asthma exacerbation | 0.97 | 0.62-1.52 | 1.27 | 0.42-3.85 | 0.93 | 0.58-1.50 | 2.19 | 0.67-7.23 | 0.88 | 0.54-1.44 |
| - Moderate asthma exacerbation | 0.98 | 0.86-1.12 | 1.08 | 0.81-1.44 | 0.97 | 0.84-1.12 | 0.94 | 0.55-1.62 | 0.99 | 0.86-1.13 |
| **30 day risk window** |  |  |  |  |  |  |  |  |  |  |
| - Severe asthma exacerbation | 0.99 | 0.62-1.61 | 1.04 | 0.35-3.04 | 0.88 | 0.55-1.41 | 0.76 | 0.06-9.67 | 1.00 | 0.61-1.65 |
| - Moderate asthma exacerbation | 0.89 | 0.77-1.04 | 1.11 | 0.84-1.46 | 0.97 | 0.84-1.12 | 1.05 | 0.52-2.12 | 0.89 | 0.76-1.04 |
| **90 day risk window** |  |  |  |  |  |  |  |  |  |  |
| - Severe asthma exacerbation | 0.96 | 0.64-1.44 | 1.05 | 0.36-3.08 | 0.89 | 0.55-1.42 | 2.23 | 0.81-6.13 | 0.88 | 0.56-1.37 |
| - Moderate asthma exacerbation | 1.00 | 0.88-1.13 | 1.10 | 0.83-1.45 | 0.97 | 0.85-1.12 | 0.71 | 0.44-1.15 | 1.03 | 0.90-1.17 |

Sensitivity analyses excluding patients: hospitalised within the risk window; smokers >40 years of age; cases unmatched on age; and a complete case analysis; varying risk window duration. IRR=incidence rate ratio. Adjusted for asthma medication use in the 90 days prior to the index date; respiratory tract infection in the 90 days prior to the index date; hospitalization for asthma in the year prior to the index date; type of CVD medicine use in the year prior to the index date; exact age; smoking status; body mass index; social deprivation; Charlson comorbidity index; and primary care asthma review in the year prior to the index date.
