# Supplementary material for: Respiratory effect of beta-blockers in people with asthma and cardiovascular disease: population-based nested case control study
Source: BMC Med. 2017 Jan 27;15:18. doi: 10.1186/s12916-017-0781-0 (PMC5270217; doi:10.1186/s12916-017-0781-0)
Supplement: Additional file 4: Table S4. — Sensitivity analyses for non-selective beta-blocker exposure and asthma exacerbations. (DOCX 17 kb) [file 12916_2017_781_MOESM4_ESM.docx]

**ADDITIONAL FILE 4: BETA-BLOCKERS IN PEOPLE WITH ASTHMA AND CVD**

**Table S4. Sensitivity analyses for non-selective beta-blocker exposure and asthma exacerbations.**

|  | **Any**  **exposure** | | **High dose**  **exposure** | | **Low-moderate**  **dose exposure** | | **Acute**  **exposure** | | **Chronic**  **exposure** | |
| --- | --- | --- | --- | --- | --- | --- | --- | --- | --- | --- |
|  | **IRR** | **95% CI** | **IRR** | **95% CI** | **IRR** | **95% CI** | **IRR** | **95% CI** | **IRR** | **95% CI** |
| **Inhaled corticosteroids by dose** |  |  |  |  |  |  |  |  |  |  |
| - Severe asthma exacerbation | 1.70 | 0.53-5.50 | 12.06 | 1.02-142.52 | 1.22 | 0.32-4.66 | - | - | 1.76 | 0.54-5.71 |
| - Moderate asthma exacerbation | 1.41 | 0.95-2.08 | 2.67 | 1.08-6.62 | 1.24 | 0.80-1.91 | 5.19 | 1.85-14.59 | 1.19 | 0.78-1.84 |
| **Hospitalised in risk window** |  |  |  |  |  |  |  |  |  |  |
| - Severe asthma exacerbation | 1.40 | 0.35-5.58 | 14.28 | 1.14-178.17 | 0.84 | 0.16-4.36 | - | - | 1.72 | 0.52-5.69 |
| - Moderate asthma exacerbation | 1.46 | 0.97-2.20 | 2.83 | 1.13-7.11 | 1.27 | 0.80-2.00 | 6.44 | 1.71-24.32 | 1.30 | 0.84-2.01 |
| **Smokers over 40 years** |  |  |  |  |  |  |  |  |  |  |
| - Severe asthma exacerbation | 1.62 | 0.49-5.33 | 22.29 | 1.10-450.94 | 1.13 | 0.29-4.39 | - | - | 1.67 | 0.50-5.54 |
| - Moderate asthma exacerbation | 1.46 | 0.97-2.19 | 2.72 | 0.99-7.46 | 1.31 | 0.84-2.04 | 7.67 | 2.46-23.95 | 1.21 | 0.77-1.89 |
| **Unmatched on age** |  |  |  |  |  |  |  |  |  |  |
| - Severe asthma exacerbation | 1.63 | 0.50-5.31 | 12.53 | 1.03-152.87 | 1.16 | 0.30-4.45 | - | - | 1.44 | 0.46-4.48 |
| - Moderate asthma exacerbation | 1.37 | 0.92-2.04 | 2.82 | 1.14-7.02 | 1.19 | 0.76-1.85 | 5.14 | 1.82-14.55 | 1.15 | 0.74-1.79 |
| **Complete case analysis** |  |  |  |  |  |  |  |  |  |  |
| - Severe asthma exacerbation | 1.27 | 0.32-4.95 | 18.30 | 1.06-315.49 | 0.76 | 0.15-3.87 | - | - | 1.36 | 0.34-5.40 |
| - Moderate asthma exacerbation | 1.48 | 0.99-2.22 | 3.59 | 1.41-9.13 | 1.26 | 0.81-1.97 | 4.86 | 1.72-13.76 | 1.27 | 0.82-1.98 |
| **30 days risk window** |  |  |  |  |  |  |  |  |  |  |
| - Severe asthma exacerbation | 0.58 | 0.07-4.78 | - | - | 0.59 | 0.07-4.84 | - | - | 0.60 | 0.07-4.97 |
| - Moderate asthma exacerbation | 1.37 | 0.88-2.15 | 2.89 | 1.07-7.79 | 1.20 | 0.73-1.98 | 3.04 | 0.81-11.43 | 1.27 | 0.79-2.05 |
| **90 day risk window** |  |  |  |  |  |  |  |  |  |  |
| - Severe asthma exacerbation | 1.40 | 0.44-4.43 | 9.11 | 0.87-95.57 | 1.01 | 0.27-3.76 | - | - | 1.46 | 0.46-4.68 |
| - Moderate asthma exacerbation | 1.28 | 0.87-1.88 | 3.36 | 1.43-7.93 | 1.09 | 0.71-1.67 | 3.76 | 1.39-10.17 | 1.12 | 0.73-1.71 |

Sensitivity analyses excluding patients: hospitalised within the risk window; smokers >40 years of age; cases unmatched on age; and a complete case analysis; varying risk window duration. IRR=incidence rate ratio. Adjusted results presented. IRR = Incidence Rate Ratios. Adjusted for asthma medication use in the 90 days prior to the index date; respiratory tract infection in the 90 days prior to the index date; hospitalization for asthma in the year prior to the index date; type of CVD medicine use in the year prior to the index date; exact age; smoking status; body mass index; social deprivation; Charlson comorbidity index; and primary care asthma review in the year prior to the index date. Empty cells (-) = inestimable due to lack of corresponding beta-blocker exposure among cases and controls.
